# Clinical Features and Genetic Background of the Periodic Fever Syndrome with Aphthous Stomatitis, Pharyngitis, and Adenitis: A Single Center Longitudinal Study of 81 Patients

**DOI:** 10.1155/2015/293417

**Published:** 2015-03-04

**Authors:** Daša Perko, Maruša Debeljak, Nataša Toplak, Tadej Avčin

**Affiliations:** ^1^Department of Allergology, Rheumatology and Clinical Immunology, University Children's Hospital, University Medical Center, Bohoričeva 20, 1000 Ljubljana, Slovenia; ^2^Unit for Special Laboratory Diagnostics, University Children's Hospital, University Medical Center, Bohoričeva 20, 1000 Ljubljana, Slovenia; ^3^Faculty of Medicine, University of Ljubljana, Vrazov trg 2, 1000 Ljubljana, Slovenia

## Abstract

PFAPA syndrome is the most common autoinflammatory disorder in childhood with unknown etiology. The aim of our study was clinical evaluation of PFAPA patients from a single tertiary care center and to determine whether variations of* AIM2*,* MEFV*,* NLRP3*, and* MVK* genes are involved in PFAPA pathogenesis. Clinical and laboratory data of consecutive patients with PFAPA syndrome followed up at the University Children's Hospital, Ljubljana, were collected from 2008 to 2014. All four genes were PCR amplified and directly sequenced. Eighty-one patients fulfilled criteria for PFAPA syndrome, 50 (63%) boys and 31 (37%) girls, with mean age at disease onset of 2.1 ± 1.5 years. Adenitis, pharyngitis, and aphthae were present in 94%, 98%, and 56%, respectively. Family history of recurrent fevers in childhood was positive in 78%. Nineteen variants were found in 17/62 (27%) patients, 4 different variants in* NLRP3* gene in 13 patients, and 6 different variants in* MEFV* gene in 5 patients, and 2 patients had 2 different variants. No variants of clinical significance were found in* MVK* and* AIM2* genes. Our data suggest that PFAPA could be the result of multiple low-penetrant variants in different genes in combination with epigenetic and environmental factors leading to uniform clinical picture.

## 1. Introduction

Periodic fever syndromes (PFSs) are a wide group of autoinflammatory disorders, characterized by recurrent and seemingly unprovoked multisystemic inflammation in the absence of infection and autoantibodies formation [1, 2]. Most hereditary PFSs are due to mutations in genes coding molecules of pyrin and tumor necrosis factor superfamilies both of which are directly involved in innate immunity [3].

Periodic fever with aphthous stomatitis, pharyngitis, and adenitis (PFAPA) represents the most common periodic fever syndrome of childhood with unknown actual incidence [4]. In contrast to monogenic periodic fever syndromes, the genetic defects in PFAPA have not yet been identified. This clinical entity is characterized by a sudden onset of fever which lasts for 3 to 6 days and spontaneously resolves afterwards. Fever is accompanied by aphthous stomatitis, pharyngitis, and cervical adenitis [5–7] with less common symptoms such as headache, rash, and gastrointestinal disturbances [5, 8]. PFAPA episodes recur every 3 to 8 weeks. During the remission, patients are asymptomatic and show normal growth and development. Disease onset is usually before the age of five and generally resolves before puberty with no consequences for the patient [7–9]. No specific diagnostic test for PFAPA is currently available; therefore, the diagnosis is based only on diagnostic criteria [10].

Despite the fact that PFAPA is considered as a sporadic disease, many studies suggest that it might have a genetic origin. Syndrome has overlapping symptoms with other PFSs with known genetic cause [11] and, furthermore, recurrent fever in the family members of PFAPA patients has been reported in several studies [10, 12]. In addition, genetic variants that are known to cause other autoinflammatory syndromes have been found in PFAPA patients [9, 13], but the impact of these genetic variants in PFAPA syndrome is still unknown.

Although the pathogenesis of PFAPA remains poorly understood, it is assumed that an environmental trigger in the context of immune abnormalities of the host may trigger disease episodes through activation of innate immune system, possibly via “absent in melanoma 2” (AIM2) inflammasome in monocytes, which leads to activation and secretion of IL-1*β*, induction of Th1-chemokines, and subsequent migration of T-lymphocytes into periphery [5].

AIM2 is a 343 amino acids long interferon inducible protein which plays a role as a receptor for cytosolic DNA. It consists of two subunits: C-terminal HIN-200 domain which binds double stranded DNA and N-terminal pyrin domain which interacts with pyrin domain of apoptosis-associated speck-like protein (ASC). ASC recruits procaspase-1 to the inflammasome complex which ultimately leads to activation of cytokines (IL-1*β* and IL-18) and inflammation [14–16]. It has been shown that* AIM2* gene is overexpressed in PFAPA patients during flare compared to PFAPA patients in remission and healthy controls [5].

Given that IL-1*β* pathway is involved in the PFAPA pathogenesis, it is likely that* AIM2* gene could also play an important role in this syndrome.

The aim of our study was to evaluate clinical features and molecular genetic background in a large, well-characterized cohort of patients with PFAPA syndrome from a single tertiary care center. In particular, we aimed to analyze involvement of* AIM2* gene in the pathogenesis of PFAPA, which has not been previously tested in patients with PFSs. A second goal was to evaluate possible association of* MEFV*,* NLRP3*,* MVK,* and* AIM2* gene variants with clinical features of the syndrome.

## 2. Methods

### 2.1. Participants

Clinical and laboratory data of 81 consecutive patients with PFAPA syndrome followed up at the University Children's Hospital, Ljubljana, were collected from January 2008 to June 2014. All participants included in the study fulfilled the clinical criteria for PFAPA syndrome [8]. Ethnicity and family history of recurrent fever, tonsillectomy, and repeated tonsillopharyngitis in family members were also obtained.

Samples from 100 apparently healthy adult subjects of Slovenian ethnicity that donated their DNA for research purposes were included in the study as healthy controls.

### 2.2. Ethics Committee Approval

The parents of each child included in the study were informed about the aim of the study and asked for written informed consent for inclusion in the study. The study was approved by the Ethics' Committee of the Republic of Slovenia and was conducted according to the principles of the Helsinki Declaration.

### 2.3. Samples and Blood Work

PFAPA patients had blood work performed during routine venipuncture initially and/or at the followup visits including complete blood count (CBC), C-reactive protein (CRP), erythrocyte sedimentation rate (ESR), serum immunoglobulin levels (IgA, IgG, IgM, and IgD), antinuclear antigen antibodies (ANA), serum amyloid A (SAA), antistreptolysin O titer (ASO), classical and alternative complement pathway activity (CH50, APH50), and lymphocyte subsets.

### 2.4. DNA Isolation and Primer Selection

DNA was isolated from 62 patients with PFAPA syndrome. Peripheral blood (5 mL) for DNA isolation was taken together with blood for routine laboratory work. DNA isolation was performed using the FlexiGene isolation kit (Qiagen, Germany), according to the recommended protocol. DNA was stored at 4°C prior to further molecular analyses.

PCR primers (Eurofins MWG Operon, Germany) were designed according to the established laboratory protocol (sequences are available upon request), covering whole coding regions and intron/exon boundaries of all four genes (*MEFV, NLRP3, MVK, *and* AIM2*) including promoter, 5′UTR, and 3′UTR regions of* AIM2* gene.

### 2.5. Polymerase Chain Reaction

PCR was performed on GeneAmp PCR System 9700 (Applied Biosystems, USA) using the AmpliTaq polymerase (Applied Biosystems, USA) for the whole* NLRP3* gene, part of* MEFV *gene (exons 1, 3, 4, and 6–10) and part of* MVK* gene (exons 2–7, 9, and 11), AmpliTaq Gold polymerase (Applied Biosystems, USA) for exons 2 and 5 of* MEFV* gene, and GoTaq G2 Flexi DNA polymerase (Promega, USA) for the whole* AIM2* gene and exons 8 and 10 of* MVK* gene.

The reactions with both AmpliTaq polymerases were set up using 100 ng of double stranded DNA, 0.2 mM of each of the dNTPs, 0.4 *μ*M of primers, 1.5 mM or 1.8 mM (for AmpliTaq Gold) of MgCl_2_, 1x reaction buffer, and 0.75 U of polymerase in a final reaction volume of 25 *μ*L. 3.4 *μ*L of 40% dimethyl sulfoxide was added when AmpliTaq Gold was used. The thermocycling procedure was conducted according to the following protocol: denaturing step at 95°C for 5 min (10 min for AmpliTaq Gold) followed by 35 cycles of 94°C for 30 s, annealing step at 58°C–60°C (adjusted for each set of primers individually) for 30 s, extension step at 72°C for 40 s, and final extension at 72°C for 7 min.

The reaction with GoTaq G2 Flexi DNA polymerase was set up using 100 ng of double stranded DNA, 0.2 mM of each of the dNTPs, 0.32 *μ*M of primers, 2 mM of MgCl_2_, 1x green buffer, and 0.75 U of polymerase in a final reaction volume of 25 *μ*L. The thermocycling procedure consisted of initial denaturing step at 95°C for 2 min followed by 35 cycles of 94°C for 30 s, annealing step at 60°C or 61°C (adjusted for each set of primers individually) for 30 s, extension step at 72°C for 40 s, and final extension at 72°C for 7 min. The PCR products were subjected to electrophoresis in 1.5% agarose gel stained with SYBR Green and photographed under ultraviolet light using the UV G:BOX (Syngene, USA).

### 2.6. Sanger Sequencing

PCR products (2.5 *μ*L) were purified using 1 *μ*L ExoSAP-IT enzyme mix (Affymetrix, USA), (incubation at 37°C for 15 min; enzyme deactivation at 80°C for 15 min) and subjected to direct nucleotide sequencing using the Big Dye Terminator cycle sequencing kit (Applied Biosystems, USA) following the manufacturer's instructions. The sequencing was performed on Applied Biosystems 3500 Genetic Analyser (Applied Biosystems, USA). Sequences were analyzed with the Sequencing Analysis Software v5.2.0 (Applied Biosystems, USA) and examined using the Nucleotide BLAST program.

All identified mutations/variants were validated in additional independent round of PCR and once again sequenced.

### 2.7. Genotyping PCR

The TaqMan (Applied Biosystems, USA) genotyping assays were used for two genetic variants that showed difference between frequency in PFAPA population and frequency in European or global population (MAF).

Genotyping experiments consisted of 10 ng of double stranded DNA, 1x Master mix, and 0.5x of specific assay in a final reaction volume of 10 *μ*L and were run on the 7500 Fast RT-PCR system. The results were analyzed with the 7500 Software v2.0.1 (Applied Biosystems, USA).

### 2.8. Statistical Analysis

Descriptive statistics were reported as means ± SD with ranges between extreme values added. Observed and expected frequencies of genetic variants were compared by using *χ* ^2^ test.

## 3. Results

### 3.1. Clinical Characteristics

In the 7-year study period, 81 patients fulfilled criteria for the PFAPA syndrome.  Table 1 describes the demographic and clinical characteristics of all PFAPA patients. The cohort consisted of 50 (63%) boys and 31 (37%) girls, with mean age at disease onset of 2.1 ± 1.5 years. Only 3 patients were older than 5 years at disease onset including twin sisters. Mean duration of febrile episode was 4.2 days with 4-week interval between episodes. The longest afebrile period was 2 months in 2 patients.

All patients were asymptomatic during the afebrile period and showed normal growth and development.

### 3.2. Signs and Symptoms

**Figure 1:**
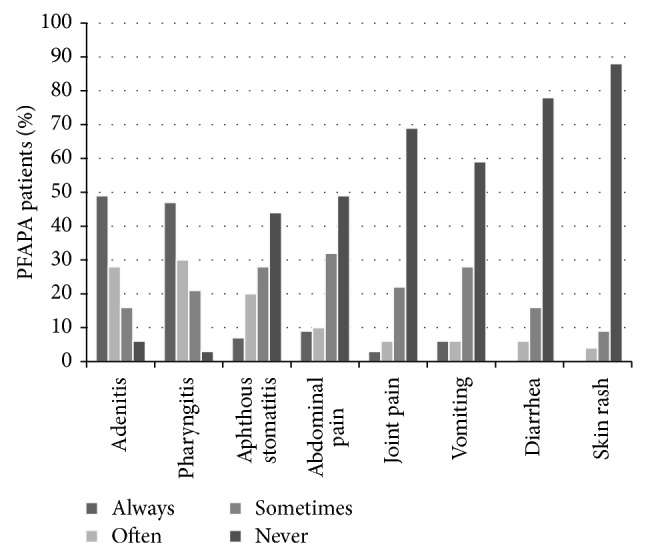
Frequencies of 8 clinical manifestations in 81 PFAPA patients.

Table 2 describes three main clinical manifestations (adenitis, pharyngitis, and aphthous stomatitis) and other signs accompanying fever in PFAPA patients. In three (4%) children only one main symptom was present, 35 (43%) patients had 2 symptoms, and 43 (53%) had all 3 main symptoms present.  Figure 1 shows frequencies (always, often, sometimes, and never) of 8 clinical manifestations in 81 PFAPA patients. In majority, patients parents reported onset of febrile episodes on a weekend (usually or often in 39/45 patients, never or sometimes in 6/45 patients).

### 3.3. Family History

In our PFAPA cohort 3 pairs of siblings were included: twin brothers, twin sisters, and two brothers. Family history of patients is presented in Table 3. Fifty out of 64 (78%) patients had positive family history meaning that their first degree relatives had recurrent fevers in their childhood or tonsillectomy performed.

### 3.4. Laboratory Values

Inflammatory parameters were in all patients elevated during febrile episodes and normal during afebrile periods. Laboratory values of tested PFAPA patients are listed in Table 4.

### 3.5. Treatment

Of the 81 patients, 27 (33%) used corticosteroids (1-2 mg/kg) at the onset of the febrile attack. They received two doses in 12-hour interval. Febrile attacks were successfully stopped in all patients. In 9/27 (33%) patients the interval between the episodes was shortened after treatment with corticosteroids. Tonsillectomy was performed in 28 (35%) patients and PFAPA resolved in 26/28 (93%). Procedure was not successful in 2/28 (7%) patients, who subsequently continued to have episodes of PFAPA, although the episodes were shorter and less frequent.

### 3.6. Genetic Analyses

DNA of patients with PFAPA syndrome was analyzed for the 3 genes associated with monogenic periodic fever (*NLRP3*,* MEFV*, and* MVK*) and for the* AIM2* gene.

### 3.7. *AIM2* Gene

Whole coding region of* AIM2* gene with promoter, 5′UTR, and 3′UTR regions was analyzed in 62 patients. We identified 7 different variants: 1 novel and 6 already reported in dbSNP database (http://www.ncbi.nlm.nih.gov/SNP) with known allele frequency in general population. All variants found were in noncoding region of the* AIM2* gene. Variants are listed in Table 5.

Genotyping assay for c.-208A≥C variant, located in promoter region, was analyzed in 100 apparently healthy subjects. Variant was present in heterozygous state in 9 subjects, meaning that statistical significance between healthy subjects and PFAPA patients has not been reached (MAF case/control = 0.06/0.045, *P* > 0.05).

### 3.8. *NLRP3* Gene

All variants listed here have codon number according to the number of the second methionine (Met) residue. We analyzed all coding regions of* NLRP3* gene in 62 PFAPA patients and identified 4 different variants of possible clinical significance in 13 (21%) patients. Variant Q703K in exon 3 was found in 9 patients; S726G in exon 4 in 1 patient; P340P in exon 3 in 1 patient; and a novel variant P200T in exon 3 in 2 patients. Q703K variant (rs35829419) has frequency 0.058 in European population (MAF). We analyzed 100 apparently healthy subjects with genotyping assay for Q703K variant. Variant was present in heterozygous state in 12 (12%) subjects meaning that statistical significance has not been reached (MAF case/control = 0.072/0.06, *P* > 0.05).

Variant S726G is a rare polymorphism (rs147946775) with MAF 0.002 in European population. Exon 4 of* NLRP3 *gene was analyzed also in this patient's parents and brother who are also carriers of this variant. Variant P200T, found in two unrelated patients, is a novel variant, but it is considered as a polymorphism according to Mutation Taster prediction program (http://www.mutationtaster.org/).

### 3.9. *MEFV* Gene

All coding regions of the* MEFV* gene were determined in 62 PFAPA patients. Six different variants were found in 5 (8%) patients, namely, E148Q and A289V in exon 2, P369S and R408Q in exon 3, I591T in exon 9, and K695R in exon 10. All variants found were in a heterozygous state. One girl carried two variants, namely, P369S and R408Q in exon 3, and one boy was found to have I591T variant in* MEFV* gene and Q703K in* NLRP3* gene. Variant with unknown significance R202Q in exon 2 was found in 24 (39%) patients in heterozygous state and in 6 (10%) patients in homozygous state.

### 3.10. *MVK* Gene

All coding regions of* MVK* gene were determined in 29 PFAPA patients and no variants of clinical significance have been identified.

In total, 17/62 patients (27%) carried 19 variants in* MEFV* and* NLRP3* genes. Other variants found in* NLRP3*,* MEFV,* and* MVK* genes were polymorphisms with presumably no clinical significance and were present in general population in high frequency.

### 3.11. Genetic Associations with Clinical Features of the Syndrome

Patients who carry one of the variants experienced disease onset at a younger age compared to those without a variant present (mean 2.1 ± 1.5 years versus 2.3 ± 1.6 years) but had been diagnosed with PFAPA after a longer period of time (mean 2.6 ± 2.5 years versus 1.8 ± 1.5 years). Duration of the febrile episode was slightly shorter in patients with a variant (mean 4.1 ± 1.6 days versus 4.5 ± 1.6 days) and the interval between the episodes was longer (mean 4.2 ± 1.8 weeks versus 3.8 ± 0.9 weeks).

In total, 63% of patients with a variant presented with all 3 main PFAPA symptoms compared to 49% of patients without a variant. No difference was found in comparing positive family history (78% versus 80%).

## 4. Discussion

PFAPA syndrome is the most common PFS of childhood with still unclear genetic background. So far no specific diagnostic test or laboratory markers are available for PFAPA and the diagnosis is based merely on diagnostic criteria and exclusion of other PFSs [10]. Since PFAPA syndrome has many overlapping features with monogenic PFSs, it has been suggested that it might have also a genetic origin. In this study, we clinically evaluated 81 PFAPA patients; performed analysis of* AIM2*,* MEFV*,* NLRP3,* and* MVK* genes in 62 patients; and assessed possible genetic correlations with clinical features.

We report a large cohort of PFAPA patients longitudinally followed for almost 7 years in a single tertiary center. Males were the majority (63%), which is similar to previous studies [6, 8, 17]. During the fever episode adenitis and pharyngitis were present in most of the patients (94% and 98%, resp.) and aphthous stomatitis was present in 56% of the patients, which is similar to other studies [5–8, 17–19]. All three major PFAPA symptoms were present in 53% of patients. This result is higher comparing to published data reporting the occurrence of all three symptoms in 28% of patients [17]. Seventy-nine percent of our patients were tested for ASO titer. Among them, 87% had negative ASO titers indicating that they did not encounter streptococcal infection in the past. To the best of our knowledge, there are no published data about ASO titers in PFAPA patients so far. According to our experience ASO titer could be a very helpful tool in differentiating PFAPA patients from patients suffering from recurrent streptococcal infections with similar clinical picture.

Optimal treatment for patients with PFAPA is uncertain and considering unknown etiology of the disease it is only symptomatic [20]. Given the fact that PFAPA is self-limiting disease, treatment of any kind is still a matter of debate. Corticosteroid therapy usually aborts an episode within hours, but it does not prevent future fever flares [3, 6]. Thirty-three percent of patients in our cohort were receiving corticosteroids at the beginning of febrile attacks with successful abortion of the attack in all of them.

Tonsillectomy in PFAPA patients is still controversial considering that affected children usually recover spontaneously after few years and the procedure itself is invasive with unknown long term effect for the patients. However, the method is effective and usually completely stops the attacks [21]. In our cohort 37% of patients underwent tonsillectomy with complete remission in 93% of them. This finding is similar to those reported in other studies where complete remission was up to 100% [19, 21].

PFAPA is classically considered as a sporadic and noninherited syndrome [8]; however, several studies reported PFAPA patients with family members having recurrent fever in their childhood [11, 12, 22]. In our cohort, we identified 3 pairs of siblings. Moreover, positive family history was found in 50/64 (78%) patients, which is the highest rate ever reported. Additionally, 21/64 (37%) of our patients had more than one first degree family member affected, clearly indicating possible familial association. In a recent study positive family history (recurrent fever) has been reported in 38/84 (45%) affected patients; additionally, 10 of these patients had a family member with diagnosed PFAPA syndrome [10]. In another study authors compared PFAPA patients with complete remission before puberty (mean duration of symptoms: 6.3 years) and those with remission after 18 years. Family history in the first and the second groups was positive in 40% and 4%, respectively [18]. Although no causative gene has been identified for the PFAPA syndrome, these findings clearly suggest genetic background rather than sporadic background. Alternatively, affected family members could be exposed to the same infection or any other environmental trigger which could ultimately lead to the development of clinical manifestations of PFAPA syndrome.

The cause and etiology of PFAPA is still unknown. Due to a sudden response to a single dose of corticosteroids PFAPA symptoms may be caused by dysregulated inflammatory cytokines production rather than by infection [23]. Studies on cytokines showed rapid rise and fall of proinflammatory cytokines IL-1*β*, IL-18, TNF-*α*, and IFN-*γ* in the early hours of flare and rise of IL-6 in the later state of the flare, with the normalization in the afebrile state of the disease [24], whereas T-cell associated IL-7, IL-17 [24], and anti-inflammatory cytokines IL-10 and IL-4 were decreased during flare [1].

IL-1*β* plays an important role in all autoinflammatory syndromes including PFAPA. Recent study strongly suggests that IL-1*β* monocyte production is dysregulated in patients with PFAPA syndrome as a consequence of alteration in inflammasome activation probably linked to genetic defect in inflammasome related genes [9]. Additionally, increased expression of IL-1 and inflammasome associated genes (*AIM2*,* CASP1*) was reported during PFAPA flare. Furthermore, therapeutic efficacy of IL-1*β* blockade on resolution of the disease flare was reported [5]. All these findings strongly indicate involvement of inflammasome related genes in PFAPA pathogenesis.

Therefore, we analyzed two inflammasome genes, whole coding region of* AIM2* gene with promoter, 5′UTR, and 3′UTR regions included, and* NLRP3* gene in 62 patients. In the* AIM2* gene 7 variants had been identified, 1 novel and 6 already reported, all located in noncoding region of the gene. Five of these variants had in our PFAPA cohort similar or smaller allele frequency compared to general population and it is less likely to have a significant role in the PFAPA pathogenesis according to* in silico* analyses we performed. Variant c.-208A≥C (rs41264459) is located in the promoter region of the gene; therefore, it could have impact on gene level expression. MAF of this variant in the general population is 0.03. In our study, we obtained MAF 0.06 in the patient cohort and MAF 0.045 in the healthy control group, which were not statistically significantly different. Although no connection was identified between the* AIM2* gene variants and PFAPA syndrome, it is still possible that epigenetic or somatic alterations of the gene could play a role in the disease onset.

Of the 62 PFAPA patients, 13 (21%) carried one of the four different* NLRP3 *variants identified in this cohort. Mutations in* NLRP3* gene are known to cause cryopyrin-associated periodic fever syndromes (CAPS).* NLRP3* gene encodes cryopyrin, a component of the NLRP3 inflammasome that activates caspase-1 leading to inflammation by excessive production of IL-1*β* [15, 25]. The most common variant in our cohort was Q703K, which was found in 9 patients (MAF 0.072) and in 12 apparently healthy individuals (MAF 0.06). Similar rates were recently reported in a study of 57 PFAPA patients in which the Q703K variant was found in 9 patients [9]. Role of this variant in CAPS syndrome is still unclear; it is considered as either a low-penetrance mutation or a polymorphism without functional effect [26]. Although statistical significance has not been reached, this does not mean that Q703K variant does not have an effect on inflammasome activation [9]. A recent study showed that the Q703K variant is a gain-of-function alteration leading to an overactive NLRP3 inflammasome [27]. Variant S726G which is found in one of our PFAPA patients is a rare polymorphism that was previously described in two Turkish patients with typical CAPS phenotype [28]. This variant is listed in the* Infevers* database (http://fmf.igh.cnrs.fr/ISSAID/infevers/) as a symptomatic. Mother and a brother of our patient are both carriers of this variant but did not exhibit clinical manifestations of PFAPA, so we suspect that it is not disease causing. P340P which is found in one patient is a synonymous variant, listed in the* Infevers* database and in HGMD database (http://www.hgmd.org/) as a disease causing mutation, but with unknown functional effect.

Variant c.598C>A which changes aminoacid proline at position 200 into threonine (P200T) was found in two unrelated patients. It is a novel variant and is considered as a polymorphism according to Mutation Taster prediction program (http://www.mutationtaster.org/). The analysis with the Variant Effect Predictor (http://www.ensembl.org/Homo_sapiens/Tools/VEP) did not provide any additional information on how this variant could influence the function of the* NLRP3* gene. Unfortunately, DNA from the parents of either patient was not available for further genetic analysis to see whether this variant was inherited or occurred* de novo*. Nevertheless, the presence of this variant in 2 patients indicates its importance in PFAPA phenotype.

Total of 5 (8%) patients carried 6 different variants in* MEFV* gene, namely, E148Q, A289V, P369S, R408Q, I591T, and K695R. All variants found are listed in* Infevers* database as variants found in patients with familial Mediterranean fever (FMF). However, due to the absence of functional tests, the consequences of these variants on* MEFV* gene function and structure remain unknown. The role of E148Q variant is uncertain, whether it is mildly pathogenic with reduced penetrance or a variant without the causative role [9, 29]. K695R and P369S variants have also been reported as having reduced penetrance [30].

Recent study on predominant mutations in* MEFV, CARD15/NOD2*,* TNFr1A, *and* NLRP3 *genes in patients diagnosed with PFAPA syndrome showed that 19 of 57 patients carried one or more of the variants tested, with* MEFV *gene variants being found in 16 patients [13]. In another study authors evaluated variants in 4 autoinflammatory genes (*NLRP3, MEFV, TNFRSF1A, *and* MVK*) in total of 57 patients, where they identified variants in 15 patients, with* NLRP3* variants found in 12 patients [9]. In spite of the high frequency of these variants, authors could not conclude whether these variants are associated with PFAPA or not.

## 5. Conclusions

PFAPA is classically considered as a sporadic and noninherited syndrome, but extremely high rate of positive family history in our cohort clearly indicates possible genetic background of the disease. Total of 17 patients (27%) carried variants in* MEFV* and* NLRP3* genes. These are all probably low-penetrant variants which are usually not confirmative for FMF or CAPS but could play a role in susceptibility to autoinflammation and as such they might also play a role in the pathogenesis of PFAPA. We hypothesize that PFAPA could be the result of multiple low-penetrant gene variants in different genes in combination with epigenetic and environmental factors leading to one uniform clinical picture.

## Figures and Tables

**Table 1:** Demographic and clinical characteristics of PFAPA patients.

Total number of patients	81
Male	50 (63%)
Female	31 (37%)
Age at disease onset (mean ± SD, range)	2.1 ± 1.5 years (0.1–6.5 years)
Age at diagnosis (mean ± SD, range)	3.9 ± 1.7 years (1.2–7 years)
Interval between disease onset and diagnosis (mean ± SD, range)	1.9 ± 1.5 years (0.4–6.5 years)
Duration of febrile episode (mean, range)	4.2 days (2–11 days)
Interval between episodes (mean, range)	4 weeks (14–60 days)

**Table 2:** Signs and symptoms present in 81 PFAPA patients.

Symptom	*n* (%)
Adenitis	76 (94%)
Pharyngitis	79 (98%)
Aphthous stomatitis	45 (56%)

Abdominal pain	41 (51%)
Joint pain	25 (31%)
Vomiting	33 (41%)
Diarrhea	18 (22%)
Skin rash	10 (12%)

**Table 3:** Family history.

Recurrent fever or/and tonsillectomy in first degree relative	50/64 (78%)

Recurrent fever or/and tonsillectomy in first degree relative, *one family member affected only *	29/64 (45%)
Recurrent fever or/and tonsillectomy in first degree relative, *more than one family member affected *	21/64 (38%)

Tonsillectomy in first degree relative	36/64 (56%)
Recurrent fever without tonsillectomy in first degree relative	14/64 (22%)

Tonsillectomy in second degree relative only^*^	6/64 (9%)
Negative	8/64 (13%)
Unknown	17/81 (21%)

^*^Excluding those with recurrent fever or tonsillectomy in first degree relative.

**Table 4:** Laboratory parameters of PFAPA patients.

Laboratory parameter	Mean ± SD (range)	
IgA	1.20 ± 0.60 g/L (0.29–3.03 g/L)	
IgG	10.18 ± 2.41 g/L (2.4–17.7 g/L)	
IgM	0.97 ± 0.35 g/L (0.35–1.76 g/L)	

Laboratory parameter		*n* (%)

IgD	<100 IE/mL	39/48 (81%)
	>100 IE/mL	9/48 (19%)
Antinuclear antigen antibodies	Negative	41/41 (100%)
Lymphocyte subsets	Normal	28/40 (70%)
	Elevated	10/40 (25%)
	Low	2/40 (5%)
Antistreptolysin O titer	ASO < 52 units/mL	44/64 (69%)
	52 < ASO < 500 units/mL	12/64 (19%)
	500 < ASO < 1000 units/mL	6/64 (9%)
	ASO > 1000 units/mL	2/64 (3%)

**Table 5:** The list of all genetic variants of the PFAPA patients detected in *AIM2* gene.

Variant	rs number	Number of patients (variant in heterozygous state)	MAF global/MAF patients
c.-536C>T	rs116289675	1	0.002/0.008
c.-605C>A	New	1	NA/0.008
c.-607delA	rs3834102	5	0.1152/0.04
c.-208A>C	rs41264459	10 + 1 homozygous	0.028/0.097
c.-28A>G	rs2298803	5	0.156/0.04
c.-21+45G>C	rs41264457	5	0.156/0.04
c.-20−43C>T	rs74555135	3	0.139/0.024

Variant nomenclature based on NCBI reference sequence NM_004833.1; MAF: minor allele frequency; NA: not available.
